# A Temporal Interactome Atlas across RNA Viruses reveals Convergent Vulnerabilities for Antiviral Repurposing

**DOI:** 10.64898/2026.09.21.753288

**Published:** 2026-09-22

**Authors:** Tavis. J. Reed, Alicja Tadych, Olga G. Troyanskaya, Ileana M. Cristea

## Abstract

Viruses remodel host protein interactions to facilitate their replication, yet these dynamic interactomes remain largely unmapped and difficult to compare across infections. Here, to facilitate comparative virology studies, we build the Interactome and Viral Infection Dynamics (InterVir) Atlas, performing thermal proximity coaggregation to profile temporal interactome remodeling across five major RNA viral pathogens. We develop PROTEA, an AI framework that quantifies interactome homology and identifies convergent regulatory hubs. Infection-specific signatures include neuronal SHC1 rewiring associated with altered measles virus replication and dengue-specific secretase interactomes linked to cell-junction remodeling. Comparative virology analyses uncover unexpected interactome convergence between measles and flaviviruses, unobserved in protein-abundance profiling. Mapping these shared regulatory programs to drug targets allowed prioritization of compound repurposing as antivirals. Experimental testing of ten compounds demonstrated antiviral efficacy across multiple infections. Together, InterVir and PROTEA establish an open-access resource for comparative virology, facilitating the identification of conserved host vulnerabilities and drug repurposing.

## Full Text Availability

The license terms selected by the author(s) for this preprint version do not permit archiving in PMC. The full text is available from the preprint server.

